# The *lavender* plumage colour in Japanese quail is associated with a complex mutation in the region of *MLPH* that is related to differences in growth, feed consumption and body temperature

**DOI:** 10.1186/1471-2164-13-442

**Published:** 2012-08-31

**Authors:** Bertrand Bed’hom, Mohsen Vaez, Jean-Luc Coville, David Gourichon, Olivier Chastel, Sarah Follett, Terry Burke, Francis Minvielle

**Affiliations:** 1 UMR 1313 INRA/AgroParisTech, Génétique Animale et Biologie Intégrative GABI, 78352 Jouy-en-Josas, France; 2 Department of Animal and Plant Sciences, University of Sheffield, Sheffield, S10 2TN, UK; 3 UE 1295 INRA, Pôle d’Expérimentation Animale de Tours PEAT, 37380, Nouzilly, France; 4 UPR 1934 CNRS, Centre d’Etudes Biologiques de Chizé CEBC, 79360, Beauvoir sur Niort, France; 5 Present address: Division of Genetics, Department of Biology, University of Isfahan, Isfahan, Iran; 6 INRA-GABI, bat 211, Centre de Recherches de Jouy, 78352 Jouy-en-Josas, France

## Abstract

**Background:**

The *lavender* phenotype in quail is a dilution of both eumelanin and phaeomelanin in feathers that produces a blue-grey colour on a wild-type feather pattern background. It has been previously demonstrated by intergeneric hybridization that the *lavender* mutation in quail is homologous to the same phenotype in chicken, which is caused by a single base-pair change in exon 1 of *MLPH*.

**Results:**

In this study, we have shown that a mutation of *MLPH* is also associated with feather colour dilution in quail, but that the mutational event is extremely different. In this species, the *lavender* phenotype is associated with a non-lethal complex mutation involving three consecutive overlapping chromosomal changes (two inversions and one deletion) that have consequences on the genomic organization of four genes (*MLPH* and the neighbouring *PRLH*, *RAB17* and *LRRFIP1*). The deletion of *PRLH* has no effect on the level of circulating prolactin. *Lavender* birds have lighter body weight, lower body temperature and increased feed consumption and residual feed intake than wild-type plumage quail, indicating that this complex mutation is affecting the metabolism and the regulation of homeothermy.

**Conclusions:**

An extensive overlapping chromosome rearrangement was associated with a non-pathological Mendelian trait and minor, non deleterious effects in the lavender Japanese quail which is a natural knockout for *PRLH*.

## Background

Coat and plumage colours result from the interplay of a number of genes, and the variety of visible colours associated with specific mutations or combination of mutations may be used to gain an insight into underlying gene action. The available knowledge of mouse coat colour mutants is quite extensive, but little is as yet known about the genes and the mutations that cause the highly variable plumage colour of birds. Recently, causal mutations for quail plumage colours *yellow*[1], *recessive black*[2], and *roux*[3] have been characterized and found to be homologous to known coat colour mutations in mice. Increasing our knowledge of colour mutations common to mammals and birds should make it possible to compare more systematically colour genes and their associated phenotypic effects in these two animal classes. Indeed, the comprehensive phenotyping of mutants, which is a common practice in mice [4] for example, has begun in quail [5,6], but more contributions are needed to enable more extensive comparisons between mammals and birds.

Until now, all the reported causal mutations in *MLPH* (*melanophilin*) of humans, mice and other species have been single-base substitutions or small deletions, the effects of which were limited to the dilution of hair [7,8] or feather [9] colour. It was suggested [10] that MLPH formed a complex with MyoVa and RAB27a for transporting melanosomes from melanocyte to keratinocyte. The *MLPH*-associated dilution of coat or plumage pigmentation should then result in the defective transport of melanosomes. This produces a *diluted*, *leaden* or *lavender* blue-grey colour and has been reported in several mammals: humans (Griscelli syndrome type 3 [7]), mice [10], cats [11], dogs [12] and minks [13]. More recently, this effect was also found in the chicken, with the identification in *MLPH* of the mutation responsible for the *lavender* plumage [9].

**Figure 1:**
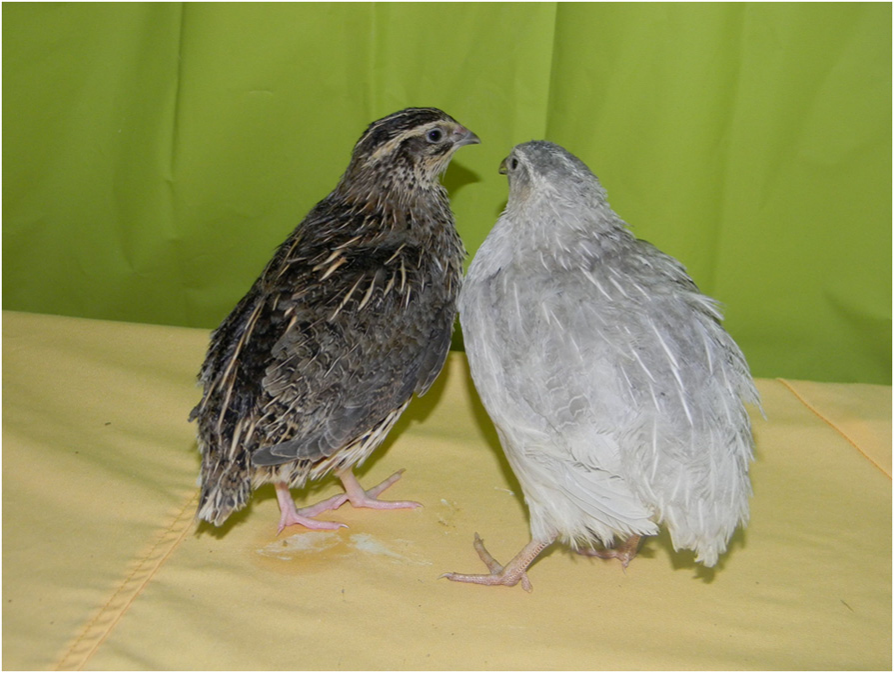
Wild-type (left) and lavender (right) Japanese quail.

In the Japanese quail (*Coturnix japonica*), the *lavender* plumage colour (Figure 1) is determined by a recessive autosomal mutation [14,15], like in chicken [16], which leads to the dilution of both eumelanin (from black to light blue-grey) and phaeomelanin (from red to buff). Intergeneric hybridisation between *lavender Gallus* and *Coturnix* has produced hybrids with a *lavender* plumage [14]. This result indicated that, as in chicken, *MLPH* was associated with the *lavender* plumage colour in quail, but did not give any information on the nature of the causal mutation. 

The primary purpose of the present study was to characterise the mutation within *MLPH* that could be responsible for the “*lavender*” plumage in Japanese quail, and to compare *lavender* and *wild-type* quail for possible phenotypic differences associated with the mutation.

## Methods

### Quail

All Japanese quail were produced and maintained at the INRA Experimental Unit PEAT in Nouzilly, France (Pôle d'Expérimentation Avicole de Tours, F-37380 Nouzilly, authorization B37-175-1, 2007) in accordance with European Union Guidelines for animal care, under authorization 37–002 delivered to D. Gourichon by the French Ministry of Agriculture. Animal procedures were approved by the Departmental Direction of Veterinary Services of Indre-et-Loire. A homozygous *lav/lav* quail line was developed in the 1980s from a single individual with *lavender* plumage given by a fancy breeder and that was crossed initially to a “wild-type” experimental line showing no visible plumage colour mutations in segregation. The line was then maintained by group mating of about 15 males and 30 females per generation. First, heterozygous *lav/+* quail were produced specifically for the experiment by crosses with the “wild-type” line. For the study of co-transmission between *MLPH* genotypes and quail phenotypes, 6 *lavender lav/lav* quail and 42 *lav/+* or *+/+* birds with wild-type plumage were obtained in a single hatch from 3 single-pair matings between non-sib heterozygous *lav/+* quail. Blood samples were collected from the six parents and the 48 progeny for DNA analysis. For the study of other possible phenotypic differences between *lavender* and wild-type animals, 130 quail (63 *lavender* and 67 wild-type) were obtained from 21 single-pair matings between *lav/lav* and *lav/+* quail, in a single hatch following a two-week egg collection period.

### DNA extraction and genomic analyses

High molecular-weight DNA was obtained from blood samples using classical DNA extraction procedure [17]. All genomic analyses were carried out using DNA samples from three *+/+* and three *lav/lav* quail. The annotation of the region was based on information from the chicken genome sequence (assembly v2.1), from the UCSC (genome.ucsc.edu) and Ensembl ( http://www.ensembl.org) genome browsers, under the assumption that the organization of chicken and quail genomes is highly similar [18]. Primers were designed using Primer3 software (frodo.wi.mit.edu/primer3/) and quail genomic sequence as a template or chicken sequence when the quail sequence was not available. When the chicken sequence was used, new quail-specific primers were designed after successful amplification and sequencing of the region. PCRs were undertaken using the Hot Start Master Mix (Qiagen). All Sanger sequencing was conducted by the Eurofins-MWG company (Ebersberg, Germany) using PCR as templates and PCR primers as sequencing primers. The list of primers used is detailed in Additional file 1: Table S1.

### Chromosome walking

To carry out this approach [19], a first PCR was completed using a biotinylated primer specific to the known region and a partly degenerate primer coupled to a unique tail sequence. After purification with stretptavidin-coated beads, a second PCR was conducted on this template using a nested primer from the known region and a primer complementary to the unique tail sequence, then the product was sequenced using these primers as sequencing primers. The primers used are detailed in Additional file 1: Table S1.

### Tissue samples and RNA extraction

Tissues (liver, dorsal skin, lung, brain and pectoral muscle) were frozen in liquid nitrogen immediately after sampling from 3 *+/+* and 3 *lav/lav* adult quail, and kept at −80 °C before use. Samples were extracted by grinding tissues in liquid nitrogen followed by re suspension in Qiazol (Qiagen). After addition of chloroform and centrifugation, the aqueous phase was recovered and supplemented with isopropanol, and total RNA was precipitated by centrifugation after ethanol washing. Quantification was performed using 1 μL of the solution in a Nanodrop (ThermoScientific), and quality (profile and RNA Integrity Number (RIN) value) controlled by analysis of 1 μL of the solution in a Bioanalyzer (Agilent).

### Gene expression

Using total RNA, RT-PCRs were performed with SuperScript II (Invitrogen) for DNA first-strand synthesis, followed by PCR using Hot-Start Master Mix (Qiagen). For *MLPH*, 6 RT-PCRs were tested, corresponding to different parts of the gene, encompassing or not the deleted region (between exons 1 to 2, 5 to 7, 1 to 7, 8 to 10, 1 to 10 and 5 to 10). For *PRLH*, 2 RT-PCRs were tested, between exons 1 to 2 and 1 to 3. For *RAB17*, 2 RT-PCRs were tested, between exons 1 to 4 and 3 to 4. Amplification was confirmed by electrophoresis on 2% agarose gel, and the sizes of amplified fragments determined by comparison to a reference ladder. The list of primers used is detailed in Additional file 1: Table S1.

### Genotyping

A PCR test was developed for genotyping the *lavender* locus in quail. A three-primer PCR test was used, with one primer in a conserved region (Gen_F) and a second primer specific to the genomic organisation of each genotype: inside the deleted region for wild-type (Gen_wt_R) and inside the inverted region for *lavender* (Gen_lav_R). Primer sequences are detailed in Additional file 1: Table S1. After PCR, amplification was confirmed by electrophoresis on 1.5% agarose gel, and the sizes of amplified fragments determined by comparison to a reference ladder. The expected sizes of PCR products were 423 bp for the band specific to wild-type genomic organization, and 630 bp for the band specific to *lavender* genomic organization.

### Husbandry and traits

Chicks were reared in group cages and given a starter diet until they were 5 weeks of age. They were sexed at 3 weeks, and males and females were kept separately thereafter. Next, they were transferred to adult individual cages in a single battery under artificial lighting for 14 hours per day, and were given a standard commercial diet. Drinking water was available at all times. All husbandry procedures followed French regulations. Individual body weights were measured weekly between the ages of 1 and 9 weeks. A 3-week feed trial was started with 96 5-month quail of both genders (48 males and 48 females) and plumage colours (52 *lavender* and 44 wild-type quail) from the 21 full-sib families. Individual feed intake during the 3-week period was registered, and body weights were measured at the beginning and the end of the test, after 12 hours’ fasting, in order to estimate body weight gain and metabolic body weight on test. For each female, the number of eggs laid during the test was recorded, and three eggs laid consecutively were weighed to estimate the total egg mass produced during the feed test. Body temperature was measured at the end of the test. Four quail did not complete the feed trial. After the feed test, all quail were fed *ad libitum* until 6 months of age when they were weighed and slaughtered at the experimental unit. Sacrificed quail were weighed before dissection (carcass body weight). Abdominal adipose tissue, right *pectoralis major* and *pectoralis minor* muscles, and liver were collected and weighed for each quail.

Blood samples were obtained from 20 *lavender* and 6 wild-type quail and plasma concentration of prolactin were measured by radioimmunoassay at the CEBC [20]. Pooled plasma samples taken from quail resulted in dose–response curves that paralleled the chicken standard curves (AFP4444B). Thus, the cross-reactivity of the chicken prolactin antibody with prolactin was equivalent in both species, and this heterologous assay could be used to assess relative levels of quail prolactin. The detection limit of the assay was 2.8 ng/ml and the intra-assay coefficient of variation was 12.5%.

### Statistical analyses

Individual growth was assessed using the nonlinear monomolecular model [21]: body weight = *A* – *B*^-*kt*^, where *A* is the asymptotic body weight, *B* is the range of body weights from hatching to asymptotic body weight, *k* is the relative rate of growth, and *t* is the age in days, and parameters of the curve were obtained by using the NLIN procedure [22]. Single curves were also adjusted for each genotype (*lav/lav* and *lav/+*) to estimate the fit (coefficient of determination) of the model. Residual feed intake (RFI), which is the part of the feed consumed that is not used for growth, maintenance or production [23] was estimated. In females, RFI was estimated for each bird as the residual (over the 21-day feed-test period) of the multiple regression of individual feed intake (FI) on body weight gain on test (BWG), metabolic body weight (MBW), and egg mass (EM). The multiple regression fitted to the data (R^2^ = 0.57) was FI = 23.3 + 1.50 BWG + 6.56 MBW +1.075 EM. To estimate individual RFI in males, the multiple regression (R^2^ = 0.72) was FI = 255.2 + 3.35 BWG + 2.91 MBW.

Parameters of individual growth curves, body weights, feed intake and carcass weight were analysed by an analysis of variance with family, sex and genotype *(lav/lav* or *lav/+*) as main effects (Full model). An analysis without the effect of genotype (Reduced model) was also run to evaluate the variation explained by plumage colour. The linear model used for the analysis of egg number and egg weight was similar but did not include the effect of sex. Body temperatures and dissection traits were analysed by an analysis of covariance, with family, sex and genotype as main effects, and with a covariable, which was the contemporaneous body weight for measures of rectal temperature and the carcass weight for dissection traits. The analyses of variance and covariance were carried out using the GLM procedure [22].

## Results

### Characterisation of the lavender mutation

**Figure 2:**
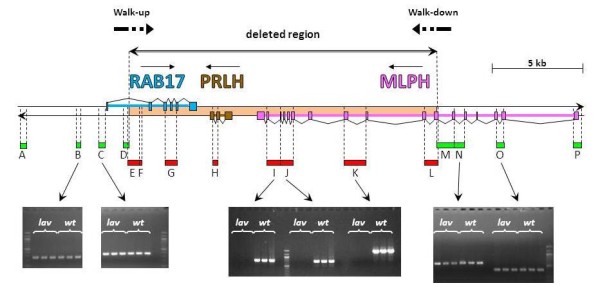
**Results of the PCR screening of the genomic region comprising *MLPH *,*PRLH *and *RAB17.*** MLPH, PRLH and RAB17 genes are coloured in purple, brown, and light blue, respectively. Exons and introns are depicted as outlined boxes and broken lines, respectively. PCR working in both *lavender* and wild-type (A-D and M-P) are in green, PCR working only in wild-type (E-L) are in red, with band visualization after gel electrophoresis for PCR B, C, I, J, K, N and O on 3 *lavender* and 3 wild-type animals. Degenerate PCR Walk-up and Walk-down are indicated by arrows.

The genomic region of *MLPH* was compared in wild-type and *lavender* Japanese quail to determine the causal mutation of the *lavender* phenotype. A preliminary screening with PCR amplification of several targets distributed over the region revealed that some consecutive PCRs failed to produce any amplification in DNA from *lavender* birds (PCR E to L, Figure 2), whereas all PCRs amplified the target region in DNA from wild-type animals. Moreover, the flanking PCRs produced the expected amplification in both wild-type and *lavender* quail (PCR A to D and M to P, Figure 2). All PCR fragments were directly sequenced from both ends and their sequences corresponded to the region expected from the chicken genomic sequence. This pattern was compatible with a deletion encompassing a large part of the *MLPH* gene, the entire *PRLH* gene and a large part of the *RAB17* gene, according to the chicken genome annotation. Additional PCR tests confirmed that a deletion was likely to be associated with *lavender* in Japanese quail, and allowed us to narrow down the two regions containing each putative breakpoint to less than 1 kb (see respectively overlapping PCRs D–E and L–M). But all attempts to close the deletion by a PCR with primers designed in the flanking regions failed, and it was concluded that the mutation was more complex than a simple deletion.

Chromosome walking from the flanking regions was used to determine the genomic structure of the region for the *lavender* allele: PCR assays were done with one primer anchored in each flanking region (corresponding respectively to regions D and M) and a degenerate one with a specific tail. After a second nested PCR, the products were sequenced directly from both ends, which revealed that a complex pattern of rearrangements had occurred to produce the *lavender* allele.

**Figure 3:**
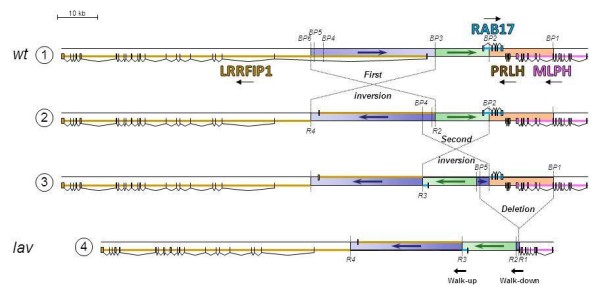
**Reconstruction of chromosomal events from wild-type genomic (1, *wt *) organization to *lavender *(4, *lav *) genomic organization.** MLPH, PRLH, RAB17 and LRRFIP1 genes are coloured in purple, brown, light blue and orange, respectively. Exons and introns are depicted as outlined boxes and broken lines, respectively. Breakpoints are numbered from BP1 to BP6 in the wild-type sequence, and recombined points are numbered from R1 to R4 in the *lavender* sequence. Degenerate Walk-up and Walk-down PCRs are indicated by arrows.

From the region upstream of the first putative breakpoint (region D), the sequences of PCR products after chromosome walking (named Walk-up on Figures 2 and 3) matched successively to two different locations in the genome. The first one (from region D, on the positive strand) was the region between the nested primer and the breaking point, followed, after the breaking point, by the second one that matched to a region 27.6 kb upstream of the breakpoint on the opposite strand.

From the region downstream of the second putative breakpoint (region M), the sequenced PCR products after chromosome walking (named Walk-down on Figures 2 and 3) matched successively to three different locations of the genome. The first one (from region M, on the negative strand) was the region between the nested primer and the breaking point, the second one was a 246-bp fragment corresponding to a region 30 kb upstream on the same negative strand, followed by the third one, matching to a region 3 kb upstream, but on the positive strand.

Figure 3 shows the most parsimonious sequence of chromosomal events that could lead from the organization of the wild-type allele (state 1) to that observed in the *lavender* allele (state 4). The organization of the genomic region for *lavender* has been constructed using the information provided by the chromosome-walking approach and complementary PCR analyses. The complex mutation spans 60 kb, and the sequence of chromosomal events consists of three ordered steps. The first one is the inversion of a 30.9-kb region upstream of *MLPH* that contains the first exon and a large part of the first intron of *LRRFIP1* (between breakpoints BP3 and BP6). The second step is another inversion of 16.4 kb overlapping the first one, which includes again a part of *LRRFIP1* intron 1 on one side, exon 1 and a large part of intron 1 of *RAB17* on the other side (between breakpoints BP4 and BP2). Finally, the last step is a 16-kb deletion overlapping the second inversion, which removes most of *RAB17*, the entire *PRLH* gene and a large part of *MLPH* (between breakpoints BP5 and BP1). BP1 is located between *MLPH* exons 9 and 10, and the only remaining genomic region is corresponding to exons 1 to 9. The breakpoints have been named in the wild-type allele according to their order along the sequence from *MLPH* (from BP1 to BP6), and named similarly in the *lavender* allele (from R1 to R4, where R stands for rearrangement). The breakpoints after the first inversion are, respectively, R4 and R2; the remaining breakpoint of the second inversion is R3 (the second one being lost during the deletion), and the scar of the deletion is R1. All the breakpoints (from BP1 to BP6 for the wild-type allele, and from R1 to R4 for the *lavender* allele) have been confirmed by specific PCR amplifications (Figure 2 and Additional file 2: Figure S1) followed by sequencing, except for BP3 and R4 for which nonspecific PCR amplification led to poor sequence quality (Additional file 1: Table S2). Larger local differences between chicken and quail genomic sequences upstream of the BP3 breakpoint are the most likely reason for this results, given that unsuccessful preliminary mapping of quail NGS reads on the chicken reference genome was already observed in this region (F. Pitel, personal communication). Sequences of wild-type and lavender breakpoint regions have been deposited into the nucleotide GenBank database, under accession numbers JX266433 to JX266439 as follows: JX266433 for BP1_wt, JX266434 for BP2_wt, JX266435 for BP4_wt, JX266436 for BP5_wt, JX266437 for BP6_wt, JX266438 for R1-R2_lav, and JX266439 for R3_lav (Additional file 1: Table S3).

Heterozygous parents *lav/+* (n = 6) and offspring (n = 48) from the informative cross were genotyped using the three-primer PCR test. The genotypes of all parents were confirmed as heterozygous, whereas the genotypes of offspring were in perfect agreement with their observed phenotypes: homozygous *lav/lav* for *lavender* birds (n = 6), heterozygous *lav/+* (n = 19) or homozygous *+/+* (n = 23) for wild-type quail.

### Gene expression

The RNA integrity number (RIN) for the extracted RNA ranged between 7.4 and 7.8. All RT-PCR amplifications were successful for *MLPH* and *RAB17* pairs of primers in liver, dorsal skin, lung, brain and pectoral muscle samples from wild-type adults. In *lavender* adults, we obtained no amplification for *RAB17* and for *MLPH* exons 1 to 10, 8 to 10 and 5 to 10, but the same products as obtained in the wild type were detected for *MLPH* exons 1 to 2, 1 to 7 and 5 to 7. No amplification was obtained for RT-PCR targeting *PRLH* in both lavender and wild-type quail, although the gene is likely to be present in wild-type birds: there are 2 chicken transcribed sequences for PRLH in GenBank (EST BX933358 / BU445468 (from ovary) and mRNA EF418015), and PRLH was reported to be present on chromosome 7 of the chicken [24].

**Association between the complex mutation revealed by the plumage colour and various traits** Parameters of the growth curves are listed and compared in Table 1. Asymptotic body weight and body weight range were influenced by the complex mutation (P < 0.05), and *lavender* quail had a consistently lighter body weight during the growth period. The relative rate of growth was similar for the two genotypes, however, indicating that the overall shape of the growth curve was not affected. Results of the feed intake trial are given in Table 2. Measures of body weight confirmed that there was only a marginal difference between genotypes (P < 0.05 or NS). Body weight gain on test was similar for the two quail groups. Daily feed intake and residual feed intake were higher (P < 0.001) for the *lavender* quail, which had a lower body temperature (P < 0.001) than wild-type quail. Egg number and egg weight were not affected. Measures taken at 6 months (Table 3) confirmed the smaller body weight (P < 0.05) and lower body temperature (P < 0.01) of *lavender* quail. Gross dissection showed that the pectoralis skeletal muscles were lighter (P < 0.001), and the liver was heavier (P < 0.001) in the *lavender* quail, whereas there was no difference in abdominal adipose tissue associated with the genotype. Plasma prolactin mean levels were not significantly different (P = 0.30) for *lavender* (82.4 ng/mL) and wild-type (54.0 ng/mL) quail.

**Table 1:** **Parameters (mean ± SD) of the growth curves**^**1**^**for 59 *lavender *(*lav/lav*) and 66 wild-type (*lav/+*) Japanese quail, and effects of the family, sex and plumage colour on the parameters of individual curves**

**Parameter of the curve**	**Plumage colour (*genotype*)**		**Percentage of the total variance explained by the model**^**2**^**(R**^**2**^**)**		**Significance of the effect of plumage colour in the Full model**
	**Lavender (*lav/lav*)**	**Wild-type (*lav/+*)**	**Full model**	**Reduced model**	
*A*	204.4 ± 32.1	215.9 ± 38.3	67	65	*
*B*	221.4 ± 34.3	233.0 ± 39.6	71	69	*
*k*	0.0405 ± 0.00604	0.0401 ± 0.00759	43	43	NS

^1^ with the monomolecular growth model: body weight = *A*-*B*^-kt^, where *A* is the asymptotic body weight, *B* is the range of body weight from hatching to asymptotic weight, *k* is the relative rate of growth, and *t* is the age in days.

^2^ The Full model includes the effects of (full-sib) family, sex and plumage colour, and the Reduced model does not include plumage colour.

*: p ≤ 0.05; NS: not significant.

**Table 2:** **Effects of family, sex and plumage colour on body weights, feed consumption, egg production and body temperature (mean ± SD) in a 3-week feed trial of 9-week old*lavender*(n = 51) and wild-type (n = 41) Japanese quail from 18 full-sib families**

**Trait**	**Plumage colour (*genotype*)**		**Percentage of the total variance explained by the model**^**1**^**(R**^**2**^**)**		**Significance of the effect of plumage colour in the Full model**
	**Lavender (*lav/lav*)**	**Wild-type (*lav/+*)**	**Full model**	**Reduced model**	
64-d BW after 12 h fasting (g)	173.3 ± 22.2	178.6 ± 23.9	75	73	*
85-d BW after 12 h fasting (g)	181.7 ± 20.1	186.5 ± 20.3	61	59	NS
Daily feed intake (g)	24.5 ± 4.3	23.0 ± 4.0	72	65	***
BW gain in 3-week feed test (g)	8.7 ± 11.7	6.7 ± 10.0	45	44	NS
Egg number^2^ on 3-week test	19.5 ± 2.0	18.4 ± 3.5	27	25	NS
Egg weight^1^ (g)	9.8 ± 0.9	9.8 ± 1.3	62	61	NS
Residual feed intake (g)	19.2 ± 33.5	−22.8 ± 24.4	44	17	***
85-d body temperature^3^ after 12 h fasting (°C)	41.02 ± 0.28	41.16 ± 0.27	60	47	***

^1^The Full model includes the effects of family, sex and plumage colour, and the Reduced model does not include plumage colour.

^2^ from 23 *lavender* and 23 wild-type females.

^3^ 85-d BW was used as a covariable for the analysis of body temperature.

* p ≤ 0.05; *** p ≤ 0.001; NS not significant.

**Table 3:** **Effects of family, sex, and plumage colour on gross body composition (mean ± SD) of 6-month old*lavender*(n = 51) and wild-type (n = 41) Japanese quail from 18 full-sib families**

**Trait**	**Plumage colour (*genotype*)**		**Percentage of the total variance explained by the model**^**1**^**(R**^**2**^**)**		**Significance of the effect of plumage colour in the Full model**
	**Lavender (*lav/lav*)**	**Wild-type (*lav/+*)**	**Full model**	**Reduced model**	
6-month body weight (g)	187.3 ± 21.1	194.3 ± 22.1	67	64	*
6-month body temperature^2^ (°C)	40.89 ± 0.35	41.03 ± 0.26	52	45	***
Carcass weight (g)	179.6 ± 19.8	187.3 ± 21.0	65	61	*
Abdominal adipose tissue^3^ (g)	4.20 ± 2.14	4.06 ± 1.99	72	71	NS
*Pectoralis* muscles^3,4^ (g)	12.7 ± 1.4	14.0 ± 1.7	83	76	***
Liver weight^3^ (g)	4.29 ± 2.03	4.10 ± 1.62	91	89	***

^1^ The Full model includes the effects of family, sex and plumage colour, and the Reduced model does not include plumage colour.

^2^ Live body weight was used as a covariable for the analysis of body temperature of 6-month fed quail.

^3^ Carcass weight was used as a covariable for the analyses of abdominal adipose tissue, breast muscle weight, liver weight, and heart weight.

^4^ Total weight of the right *pectoralis major* and *pectoralis minor* muscles.

*: p ≤ 0.05; ***: p ≤ 0.001; NS: not significant.

## Discussion

We have shown that a complex mutation was associated with the *lavender* phenotype in Japanese quail, and genotyping this mutation in an informative cross confirmed that the mutation co-segregated with the phenotype. The region affected by the complex mutation is highly conserved between birds and mammals, and *MLPH*, *PRLH*, *RAB17* and *LRRFIP1* are found in the same order and relative orientation in Zebra Finch (chromosome 7), Chicken (chromosome 7), Mouse (chromosome 1) and Human (chromosome 2). The same region can be found in the genome of the turkey, but it is distributed on several contigs because an assembly is not yet available. This conserved synteny indicates that the genes involved in the present mutation and those already studied in mammals or chicken are orthologous, and that this region is not generally affected by genomic instability.

The characterization of the detailed genomic structure of the mutation leading to the *lavender* phenotype in quail showed that an homologous phenotype between quail and chicken, demonstrated by the production of *lavender* intergeneric hybrids [14], was apparently the consequence of very different mutational events: a single base-pair substitution in chicken [9] versus a complex and large mutation with three successive chromosomal rearrangements (two inversions and one deletion), affecting four consecutive genes in quail. The reconstruction of the most likely sequence of chromosome rearrangements leading to the *lavender* allele raises the question of the molecular mechanism involved. Intermediate steps (2 and 3, Figure 3) between wild-type and *lavender* alleles were not observed in the population (PCR screening of the breakpoints, data not shown). Then, it was not possible to determine whether the rearrangements occurred as three unrelated events, with intermediate genotypes now lost or not represented in the population, or in only one step. If the three mutations occurred independently, something may have increased the probability of recurrent chromosome rearrangements in the same region. Otherwise, a complex and dramatic change in genome organization took place during a single meiosis, leading to the *lavender* allele. Screening the genomic region with chicken or quail sequence data (acquired during the study) did not reveal any particular structure, such as repeats or transposable elements that might have facilitated chromosomal rearrangements.

Very few mutations similar to the one observed here, i.e. overlapping chromosomal rearrangements, have been reported. In Human, complex chromosome rearrangements have been observed in cancer cells, and some are even characteristic of specific cancers [25], and other genetic diseases. For example, the Wiskott-Aldrich syndrome results from a complex mutation due to two deletions and one inversion in a 4.5-kb region [26]. In that example, the three chromosomal events were contiguous, but it has not been confirmed that they were overlapping, and the family study revealed that the complex mutation appeared in a single step. Similarly, another complex human mutation [27] resulted from two deletions and one inversion in a 2-Mb region, and all were contiguous. The pattern of the mutation suggested two overlapping chromosomal changes: an inversion followed by a deletion, and the family study indicated that this complex mutation occurred as a single event. These observations of similar *de novo* complex chromosomal changes suggest that the chromosomal rearrangements leading to the *lavender* quail may have also taken place in a single step.

Mechanisms proposed to explain complex mutational events combining inversion and deletion are based on strand misalignment of sequences with complementary features during DNA replication or meiosis. We have carefully checked the breakpoint regions for a common DNA pattern. These regions were not located on repeated or transposable elements, but some sequences flanking breakpoints shared a common G(C)_3-5_A pattern (Additional file 1: Table S2). Interestingly, a 2.2-kb DNA sequence (between BP4 and BP5, Figure 3) appears to be involved in all the chromosomal changes. The first inversion changed its sequence orientation, the second one restored it, and the deletion removed this DNA portion together with the region containing *RAB17*, *PRLH* and *MLPH*, leaving only a short 246-bp region (between BP6 and BP5, Figure 3) translocated 30 kb downstream by the two inversions. The sequence of this DNA segment did not show any particular structure, however.

The complex mutation found in the Japanese quail is not lethal, although it has a major impact on the DNA sequence of *MLPH*, but also on *PRLH*, which is completely missing, *RAB17*, which is mostly deleted, and to a lesser extent on *LRRFIP1*. Consequently, only expression of a truncated gene is possible for *MLPH*, and *RAB17* and *PRLH* are not expressed. This is confirmed by the RT-PCR results, with no expression of *RAB17* in *lavender*, and no transcript for *MLPH* beyond exon 9. PRLH (Prolactin Releasing Hormone) is one of the hypothalamic peptide hormones that regulate the production of pituitary hormones, and it was first identified as a prolactin-releasing factor in mammals. PRLH also acts as a neuromodulator of pituitary products, and is involved in the control of metabolism, energy homoeostasis and food intake [24]. The absence of the whole DNA region for *PRLH* in *lavender* quail was not associated with any difference in plasma prolactin concentration. Indeed, secretion of prolactin in quail is probably under the control of the vasoactive intestinal peptide (VIP), as in turkey [28] and zebra finch [29], which would explain the unchanged prolactin levels of *lavender* quail. *RAB17* is a member of the RAB family (Ras proteins involved in membrane trafficking, part of the RAS oncogene superfamily). It encodes the RAS-associated protein 17 found in epithelial cells [30] and may be involved in membrane trafficking. It is almost completely deleted in *lavender* quail, but it is possible that its loss is compensated by another gene of the same family through functional redundancy, since the RAB gene family has many members (for example, 67 protein-coding genes in humans, and 53 protein-coding genes in chicken are annotated in Homologene NCBI database: http://www.ncbi.nlm.nih.gov/homologene). *RAB17* particularly, is phylogenetically very close to *RAB5* genes (*RAB5A*, *RAB5B* and *RAB5C*) [31]. *RAB17* expression [32] is regulated by *MITF* in mammalian cell lines (melanomas) and melanocytes with *RAB17* knockdown have reduced filopodia formation, leading to impaired melanosome transfer. Then, *RAB17* might make some contribution to the quail plumage colour but it is probably not responsible for *lavender* in quail because hybrids between *lavender* chicken and quail would have the RAB17 function rescued by the normal chicken RAB17, and should have shown a wild-type, undiluted, plumage colour, which they did not [14]. *LRRFIP1* (Leucine-Rich Repeat (in Flightless I) Interacting Protein-1) encodes a protein involved in the regulation of the TLR signalling pathway [33] and the production of type-I interferon in response to pathogens [34]. This immune function has not been investigated in the present study, but it is likely that this gene was only affected marginally by the complex mutation because it lies mostly outside the deleted and inverted region of the *lavender* quail DNA sequence. Indeed, only its putative first exon was involved, whereas gene annotation in human and mouse indicates that only one alternatively spliced variant out of five described is starting from this first exon, with relatively few ESTs including this first exon.

Past results in chicken [9], mice [10], cats [11], and dogs [12] point to the large deletion in *MLPH* as the most likely cause of the “*lavender*” plumage colour in the Japanese quail. In the present study, the *lavender* genotype was also associated with slightly lower growth, as for *albino*[35], *roux*[5] and *silver*[36] mutations in quail. The percentage of the phenotypic variation of the body weight traits which was accounted for by the linear model (R^2^) did not increase by more than 3 points (Tables 1, 2 and 3) when plumage colour was included (Full model), despite statistical significance. Yet, as similar associations between lower body weight and diluted plumage colour due to various mutations were also reported in the chicken (eg: [35]), these convergent observations in quail and chicken across several independent genes for plumage colour suggest that the association may be due to a pleiotropic effect related to melanin expression. Indeed, it was found recently that the feathers of *roux*, *lavender* and *albino* quail, which have lower body weight than wild-type birds, each had lower total melanin content than wild-type and *yellow* quail [37] with similar adult body weight [6]. Further study will be needed to find the possible causes of this covariation because melanins have several suggested functions (eg: [38,39]). Most other associated phenotypic effects observed in *lavender* quail are probably not attributable to *MLPH* alone, since melanophilin defects in *MLPH*-mutated humans or mice have not been found to be associated with any noticeable phenotypic consequence other than the colour dilution [7].

The very significant differences in higher residual feed intake and lower body temperature in *lavender* quail during the feed trial (Table 2) were associated with a fair portion of the variation as R^2^ increased by 27 and 13 points, respectively, when the effect of plumage colour was included in the linear model. This phenotypic pattern might be related to central homeothermy-related deficiency induced by the effect of the complex mutation on *PRLH* as was reported in PRLH-deficient rodents [40]. Of course, other genes linked to *lavender* might also be involved in some of the physiological changes observed in this study.

## Conclusion

As in Humans, complex mutations may occur in birds, and an extensive overlapping chromosome rearrangement was associated with a non-pathological Mendelian trait and minor, non deleterious effects in the lavender Japanese quail which is a natural knockout for *PRLH*.

## Competing interests

The authors declare they have no competing interests.

## Authors’ contributions

JLC carried out the genomic analyses, the gene expression and the genotyping, and MV and SF contributed to the early genomic work. DG supervised the production of experimental birds. OC carried out the study of PRLH activity. BB and FM designed the study, and wrote the paper. TB contributed to the study and to the redaction of the paper. All authors read and approved the final manuscript.

## Supplementary Material

Additional file 1 **Table S1. **List and sequence of primers used during the study. Table S2: Sequence at breakpoints for the three chromosomal changes. Table S3: Sequences containing breakpoints uploaded in GenBank database. Click here for file

Additional file 2 **Figure S1.** Results of the confirmation PCR for lavender breakpoints R1, R2, both R1 and R2, and R3 with band visualization after gel electrophoresis. PCR containing both R1 and R2 breakpoints was performed using primers R2_F and R1_R (see Additional file 1: **Table S1**). PCR are working on all lavender samples (lav) and not on wild-type samples (wt). Click here for file

## Author details

^1^UMR 1313 INRA/AgroParisTech, Génétique Animale et Biologie Intégrative GABI, 78352 Jouy-en-Josas, France. ^2^Department of Animal and Plant Sciences, University of Sheffield, Sheffield, S10 2TN, UK. ^3^UE 1295 INRA, Pôle d’Expérimentation Animale de Tours PEAT, 37380, Nouzilly, France. ^4^UPR 1934 CNRS, Centre d’Etudes Biologiques de Chizé CEBC, 79360, Beauvoir sur Niort, France. ^5^Present address: Division of Genetics, Department of Biology, University of Isfahan, Isfahan, Iran. ^6^INRA-GABI, bat 211, Centre de Recherches de Jouy, 78352 Jouy-en-Josas, France.
