# Supplementary material for: The lavender plumage colour in Japanese quail is associated with a complex mutation in the region of MLPH that is related to differences in growth, feed consumption and body temperature
Source: BMC Genomics. 2012 Aug 31;13:442. doi: 10.1186/1471-2164-13-442 (PMC3484014; doi:10.1186/1471-2164-13-442)
Supplement: Additional file 1 — Table S1. List and sequence of primers used during the study. Table S2: Sequence at breakpoints for the three chromosomal changes. Table S3: Sequences containing breakpoints uploaded in GenBank database. [file 1471-2164-13-442-S1.docx]

Supplementary Table 1 : List and sequence of primers used during the study

| **Primer name** | **Sequence** | **Comment** |
| --- | --- | --- |
|  |  |  |
| **Primers for PCR** |  |  |
| A_F | TTTCCTCCTTGGTCTTTAACACA | PCR A (forward) |
| A_R | GATTCTGAGGATTTGTGGAACTG | PCR A (reverse) |
| B_F | AGCAAAAGCATCTGCCTCAT | PCR B (forward) |
| B_R | TTACAGTGGCACAGCAGAGG | PCR B (reverse) |
| C_F | CTCTCTTTGTCTGCCCCTTG | PCR C (forward) |
| C_R | GCCAAAGTTGAGAGGTGAGC | PCR C (reverse) |
| D_F | TCTTGCCATTCTCCTCAGGT | PCR D (forward) |
| D_R | ACCTATTTGCCCCCGTAATC | PCR D (reverse) |
| E_F | TGCAGCTTCTTGTGAAGACA | PCR E (forward) |
| E_R | CTTAGTGGCAGCAGCACATC | PCR E (reverse) |
| F_F | ACCAACTTGATGTGCTGCTG | PCR F (forward) |
| F_R | TATTGGGCGATTGGGTTAAA | PCR F (reverse) |
| G_F | GCCAGGAGAAGTATCACAG | PCR G (forward) |
| G_R | GACGTCTCCATGAAGAGCA | PCR G (reverse) |
| H_F | CACGGCCGGTGTACCAGG | PCR H (forward) |
| H_R | TGAGCGCTCCATGGAAATC | PCR H (reverse) |
| I_F | AAAGAGACGCTCCACTCTCC | PCR I (forward) |
| I_R | AGGTCCTCAGGAGGAGGTTC | PCR I (reverse) |
| J_F | GCGATTTCAAAGGGACTGTT | PCR J (forward) |
| J_R | CTACCTGACTGCTGGGAAGG | PCR J (reverse) |
| K_F | TGGAGGAGCTCATCTCTGGT | PCR K (forward) |
| K_R | TGGAGGAAAAACTCCTGGTG | PCR K (reverse) |
| L_F | CAAGCAAGCAGTGAGCTTTG | PCR L (forward) |
| L_R | TTACTCTGGCCCAGCTGAAG | PCR L (reverse) |
| M_F | GAACACAGCTGCCAGGTCA | PCR M (forward) |
| M_R | AAGATGCGCAAGACAAAGC | PCR M (reverse) |
| N_F | AATCAAAGGGGTGCACAGAC | PCR N (forward) |
| N_R | CATGGAGCTGAAGCAGAGTG | PCR N (reverse) |
| O_F | ACTCGAGGGAACCGATCTTC | PCR O (forward) |
| O_R | CTGGATTGCCGCTTCTACAC | PCR O (reverse) |
| P_F | GCTTTAAGACCGGTTGGATCTAC | PCR P (forward) |
| P_R | ACACTGACACAGCTGATAACATCAC | PCR P (reverse) |
|  |  |  |
| **Primers for chromosome walking** | |  |
| FP1 | CAGTTCAAGCTTGTCCAGGAATTCNNNNNNNGGCCT | degenerate primer 1 (forward) |
| FP2 | CAGTTCAAGCTTGTCCAGGAATTCNNNNNNNGCGCT | degenerate primer 2 (forward) |
| FP3 | CAGTTCAAGCTTGTCCAGGAATTCNNNNNNNCCGGT | degenerate primer 3 (forward) |
| FP4 | CAGTTCAAGCTTGTCCAGGAATTCNNNNNNNCGCGT | degenerate primer 4 (forward) |
| FP | CAGTTCAAGCTTGTCCAGGAATTC | tail primer (forward) |
| Walk-Up | GGTGCAATCTTCTGGGGTTA | primer for Walk-up (reverse) |
| Walk-Up-Nest | TGCAGCTTCTTGTGAAGACA | nested primer for Walk-up (reverse) |
| Walk-Down | AAGATGCGCAAGACAAAGC | primer for Walk-down (reverse) |
| Walk-Down-Nest | TGTCTCTCAGTCCTTCCCAGATTAC | nested primer for Walk-down (reverse) |
| **Primers for confirmation of lavender breakpoints** | |  |
| R1_F | GGTTTTCTCATGGCAAGCTC | PCR containing R1 (forward) |
| R1_R | AGCACCCAAACTGAATGAGG | PCR containing R1 (reverse) |
| R2_F | CCTGGCAGATGAGGATGACT | PCR containing R2 (forward) |
| R2_R | TCTGCATGATCGTTCTCCTG | PCR containing R2 (reverse) |
| R3_F | TGAAGACAGTTTGTGTTGATTACGG | PCR containing R3 (forward) |
| R3_R | AACCTGTCTCTCAAAGCAGCA | PCR containing R3 (reverse) |
|  |  |  |
| **Primers for genotyping** | |  |
| Gen_F | AGCACCCAAACTGAATGAGG | Common primer (forward) |
| Gen_lav_R | CCTGGCAGATGAGGATGACT | Primer for lavender PCR (reverse) |
| Gen_wt_R | CAAGCAAGCAGTGAGCTTTG | Primer for wild-type PCR (reverse) |
|  |  |  |
| **Primers for RT-PCR** | |  |
| MLPH_ex1_F | TAGGCTTGTCGTGCAAGATG | exon 1 MLPH (forward) |
| MLPH_ex2_R | TCTTGCTGCTTTCCTGGTCT | exon 2 MLPH (reverse) |
| MLPH_ex5_F | CACTGGGGATGACAGTGATG | exon 5 MLPH (forward) |
| MLPH_ex7_R | TTCAGCTGGGCCAGAGTAAT | exon 7 MLPH (reverse) |
| MLPH_ex8_F | CAAAGCTCACTGCTTGCTTG | exon 8 MLPH (forward) |
| MLPH_ex10_R | TCCTCCAGCTTCCTCTTCAA | exon 10 MLPH (reverse) |
| PRLH_ex1_F | CACGAAAATCCCTTTCCTGA | exon 1 PRLH (forward) |
| PRLH_ex2_R | TCTCCTTACTCCTGATTTCCA | exon 2 PRLH (reverse) |
| PRLH_ex3_R | GTACCAGGAGGGGTCGATGT | exon 3 PRLH (reverse) |
| RAB17_ex1_F | CTCTGCGACCCCTGTGTAG | exon 1 RAB17 (forward) |
| RAB17_ex3_F | CACGCTGCACTCCTTGTTTA | exon 4 RAB17 (forward) |
| RAB17_ex4_R | AACCTCCCGTTCCTCAGC | exon 4 RAB17 (reverse) |

Supplementary Table 2 : Sequence at breakpoints for the three chromosomal changes

|  | First breakpoint | Second breakpoint |
| --- | --- | --- |
| First inversion | BP3 | BP6 |
|  |  | AAGGACACCTACA / GAGGCAAACTGACCA |
|  |  | TTCCTGTGGATGT / CTCCGTTTGACTGGT |
| Second inversion | BP4 | BP2 |
|  | AAGTGTGCAATAAG / GCCCAGCAATTTCA | TCTCCAGCTTTGGT / GCCCCATGTTGCTT |
|  | TTCACACGTTATTC / CGGGTCGTTAAAGT | AGAGGTCGAAACCA / CGGGGTACAACGAA |
| Deletion | BP5 | BP1 |
|  | CCCCTTTGGCATC / TCGATGGTGCAAGCA | ACCCAAGAGATTTA / CAGCCCCCAGCACA |
|  | GGGGAAACCGTAG / AGCTACCACGTTCGT | TGGGTTCTCTAAAT / GTCGGGGGTCGTGT |

Supplementary Table 3: Sequences containing breakpoints uploaded in GenBank database

BP1_wt : sequence containing wild-type BP1 breakpoint (GenBank number JX266433)

caagcaagcagtgagctttggggctcccttcacatcctcatcggaggtgtccatatctgc

agagtagggggaccggggctgctccatccaggggctgcctgtggagggagaaagcttcag

tgtcaggacccaagagatttacagcccccagcacaaagccctggatgtcagtcccatccc

tcagacgagcaaataagcaggtcaatttactcattcattcctgcagagtagttgcagggg

ttcagcctgctgctccatccacctaaccagggtcctgctgagctacaggcaggcacaagg

aacattcccttcaccctctcagagcccaagcagctgggaccctcaccacaaccttctcat

gcctcttcaatcatattgcagcccaggtttgggcactgctcctcattcagttttgggtgc

t

BP2_wt : sequence containing wild-type BP2 breakpoint (GenBank number JX266434)

tgaagacaagttgtgttgattacgggggcaaacaggtggggaacagagaaaaattaaagg

cccgaagcccgccagattgcaggggagaaactgtgaaatgaaggggcaggcagaatgcag

aaacaaggggcttttgtaagaggagggagccacggggggctgggagaaggcatggggtgt

gaacccactgctcagggctgcactatgggagggttagattggacattgggaagaatctct

tcacagagggggtggtcagggattggaaagggctgccagcctggagtgcaaaacaccctc

acaagctctccaggggtcaggcttggggccaccactgttatcccgggctcctctctcaac

ccatgtgatagagacacactgggtggtgcaatagtcatgaggcttctccagctttggtgc

cccatgttgcttaattcctgttaaggatggtgtttgtaatgcagccggaggcacacagag

atccttgactatatctcagaaggtatgaaatctctccatagcaagccact

BP4_wt : sequence containing wild-type BP4 breakpoint (GenBank number JX266435)

tggatccttcagacagataagcaaatcacaattgttgaatctaaaatgatatccagtagg

aaaaacccaagaaaaccaaccgacagtaagagcaaacactgcactttgacaggaatataa

taaggtccacagcactgtctcctaaaacacccacttgcagtaagctgtgttcagtgatcc

cttccacttgccataatggctctgcgctctctccagcttgacatcttagtacaatgctac

acacagaaaatgaattgaactgtatttcagatccttgataatgctccgccacttctttct

agctaaagccaagctgcaaacggcatgtttcacagtagggctggtcagtaagcgcccagg

tctcggtagaccttcagtaaggaagtacacatgctaattgaattcagctgttggtacaca

tcagcaaagagcaaacacgcttgtctggaccatgctgctcatggaagaagcaagcacaat

agccatatttggagcctgcaataaacagcactattttggtggcttctttatagagtaaac

acaggtcagggagggctccagggctggatgaatgccagcacctttttgaacacggacata

aacaaagcttttgttcctgactggtaatagcatttgaaatacaaataacaactgctagtc

agagattgctacaatcaatatagtcagtgaagaccaaagcgtggtacccacagcatccca

aacttcatctgaataagtgtgcaataaggcccagcaatttcagcatgttcacatgtcaca

aatctcagcttaaagcagcacccataagcttt

BP5_wt : sequence containing wild-type BP5 breakpoint (GenBank number JX266436)

cagttcttattttgttcctcagaattccagaacttagtttttcctctgttaacttcaagt

gccaggagaacgatcatgcagacattcccctttggcatctcgatggtgcaagcacaagtt

atctctgtttaatgggtcagcattcttgctctccatgcttaacattttaagccagacacc

aagaagaggggaagaaacccatagctaatgaggaatttacataagaacagcatgaacttg

aaaaacatttgcttatgcatttgccagaaaatgcatctcagagataattcaagcatgtga

ccagaagctgggaagtctcgttgacaagaaccctttccccataagtgaagcaaatgttta

ctttgcggagctctgcagtgaacactgcgtacagctctacaacataagagacaattaagg

agttgcatcttacttcataaattgtattgccatgcagtcacaaagggctgctgctaaaaa

tacagtctaaacaataactgaactccatcatgagatcgcacggattgccaaagctcaccc

cagcacaaggaatcaatcttaacacacctgtaaaaaggggggtatcatgcaacaattgtc

atagaccctagcagcattc

BP6_wt : sequence containing wild-type BP6 breakpoint (GenBank number JX266437)

taaaccgtcaccaaggcaatggactcaagtacacaaaaacaaatgcttcttaggaaaaaa

aggcccggttttcatcacaagaacatgctgccagtatcaacagataatgttcgattgagg

ctagatggcgacacagcaaggaagagaaagaaaactgcttacaacattccacattcacta

cagacaaataaaacactgtgtcaactcaaatgtacaacagtcatttctttaccttacgta

cctgcaaacatggaagactcacgccatcaaacatcttagcagaactattaacttagacaa

gagtccaattttccaacgcttaaggacacctacagaggcaaactgaccaaaaagagagct

gttgtgcctcgtgatgcccagcagcaacctaagcatcttttcctgacttactaattcaca

ccacacaatcctgcaagaccagggtttgttgggaggttaaattaggatttctcatggcaa

gctcgtcttgcaagggcgtcccaagagcaagttcttattttgttcctcagaattccagaa

cttagtttttcctctgttaacttcaagtgc

R1-R2_lav : sequence containing lavender R1 and R2 breakpoints (GenBank number JX266438)

cctggcagatgaggatgactgcagaagagaaacctgcaggatagcaaagaaaacaagcca

cagtgaggcttgctcccgtagcaggcaaactgaccaaaaagagagctgttgtgcctcgtg

atgcccagcagcaacctaagcatcttttcctgacttactaattcacaccacacaatcctg

caagaccagggtttgttgggaggttaaattaggatttctcatggcaagctcgtcttgcaa

gggcgtcccaagagcaagttcttattttgttcctcagaattccagaacttagtttttcct

ctgttaacttcaagtgccaggagaacgatcatgcagacattcccctttggcatctcagcc

cccagcacaaagccctggatgtcagtcccatccctcagacgagcaaataggcaggtcaat

ttactcattcattcctgcagagtagttgcaggggttcagcctgctactccatccacctaa

ccagggtcctgctgagctacaggcaggcacaaggaacattcccttcaccctctcagagcc

caagcagctgggaccctcaccacaaccttctcatgcctct

R3_lav : sequence containing lavender R3 breakpoint (GenBank number JX266439)

tgaagacaagttgtgttgattacgggggcaaacaggtggggaacagagaaaaattaaagg

cccgaagcccgccagattgcaggggagaaactgtgaaatgaaggggcaggcagaatgcag

aaacaaggggcttttgtaagaggagggagccacggggggctgggagaaggcatggggtgt

gaacccactgctcagggctgcactatgggagggttagattggacattgggaagaatctct

tcacagagggggtggtcagggattggaaagggctgccagcctggagtgcaaaacaccctc

acaagctctccaggggtcaggcttggggccaccactgttatcccgggctcctctctcaac

ccatgtgatagagacacactgggtggtgcaatagtcatgaggcttctccagctttggtgc

ccagcaatttcagcatgttcacatgtcacaaatctcagcttaaagcagcacccataagct

tttaattgaggaaaaaataatccaaaagttactaatgctgcttt

Supplementary Figure 1 : Results of the confirmation PCR for lavender breakpoints R1, R2, both R1 and R2, and R3 with band visualization after gel electrophoresis. PCR containing both R1 and R2 breakpoints was performed using primers R2_F and R1_R (see Supplementary Table 1). PCR are working on all lavender samples (lav) and not on wild-type samples (wt).
