# Supplementary material for: The lavender plumage colour in Japanese quail is associated with a complex mutation in the region of MLPH that is related to differences in growth, feed consumption and body temperature
Source: BMC Genomics. 2012 Aug 31;13:442. doi: 10.1186/1471-2164-13-442 (PMC3484014; doi:10.1186/1471-2164-13-442)
Supplement: Additional file 2 — Figure S1. Results of the confirmation PCR for lavender breakpoints R1, R2, both R1 and R2, and R3 with band visualization after gel electrophoresis. PCR containing both R1 and R2 breakpoints was performed using primers R2_F and R1_R (see Additional file 1: Table S1). PCR are working on all lavender samples (lav) and not on wild-type samples (wt). [file 1471-2164-13-442-S2.pptx]

## Slide 1
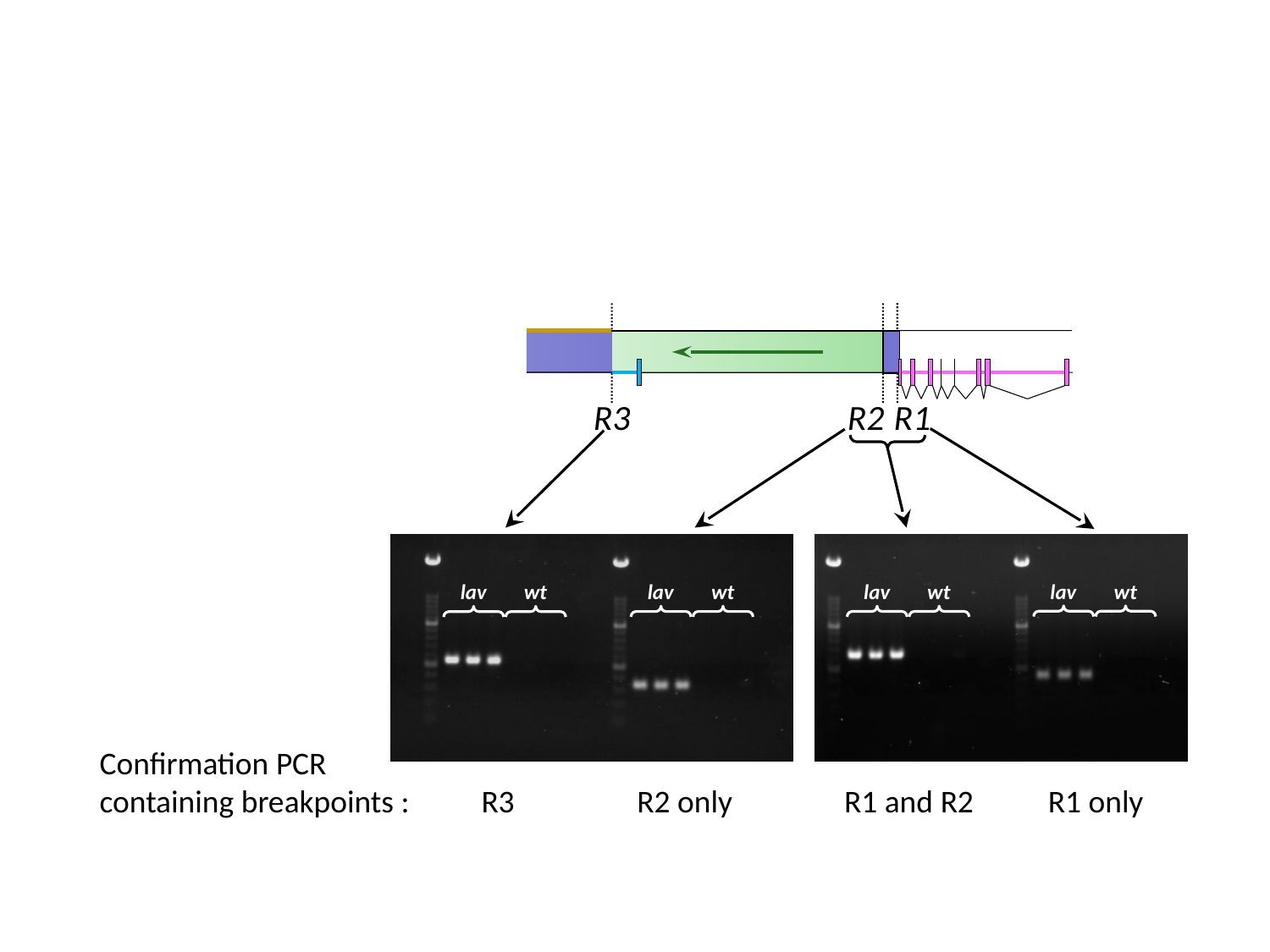

R3
R2
R1
lav
wt
lav
wt
lav
wt
lav
wt
Confirmation PCR
containing breakpoints :	R3	R2 only	R1 and R2	R1 only
